# Harnessing nanomedicine for enhanced immunotherapy for breast cancer brain metastases

**DOI:** 10.1007/s13346-021-01039-9

**Published:** 2021-10-30

**Authors:** Christine P. Carney, Nikhil Pandey, Anshika Kapur, Graeme F. Woodworth, Jeffrey A. Winkles, Anthony J. Kim

**Affiliations:** 1 Department of Neurosurgery, University of Maryland School of Medicine, Baltimore, MD 21201 USA; 2 Marlene and Stewart Greenebaum Comprehensive Cancer Center, University of Maryland School of Medicine, Baltimore, MD 21201 USA; 3 Department of Surgery, University of Maryland School of Medicine, Baltimore, MD 21201 USA; 4 Center for Vascular and Inflammatory Diseases, University of Maryland School of Medicine, Baltimore, MD 21201 USA; 5 Department of Pharmacology, University of Maryland School of Medicine, Baltimore, MD 21201 USA; 6 Department of Pharmaceutical Sciences, University of Maryland School of Pharmacy, Baltimore, MD 21201 USA; 7 Department of Surgery and Neurosurgery, University of Maryland School of Medicine, 800 West Baltimore St., Baltimore, MD 21201 USA; 8 Departments of Neurosurgery, Pharmacology, and Pharmaceutical Sciences, University of Maryland School of Medicine, 655 W Baltimore St., Baltimore, MD 21201 USA

**Keywords:** Breast cancer brain metastases, Nanoparticles, Nanotechnology, Immunotherapy, Nanoimmunotherapies, Immune checkpoint inhibitors, Blood–brain barrier

## Abstract

Brain metastases (BMs) are the most common type of brain tumor, and the incidence among breast cancer (BC) patients has been steadily increasing over the past two decades. Indeed, ~ 30% of all patients with metastatic BC will develop BMs, and due to few effective treatments, many will succumb to the disease within a year. Historically, patients with BMs have been largely excluded from clinical trials investigating systemic therapies including immunotherapies (ITs) due to limited brain penetration of systemically administered drugs combined with previous assumptions that BMs are poorly immunogenic. It is now understood that the central nervous system (CNS) is an immunologically distinct site and there is increasing evidence that enhancing immune responses to BCBMs will improve patient outcomes and the efficacy of current treatment regimens. Progress in IT for BCBMs, however, has been slow due to several intrinsic limitations to drug delivery within the brain, substantial safety concerns, and few known targets for BCBM IT. Emerging studies demonstrate that nanomedicine may be a powerful approach to overcome such limitations, and has the potential to greatly improve IT strategies for BMs specifically. This review summarizes the evidence for IT as an effective strategy for BCBM treatment and focuses on the nanotherapeutic strategies currently being explored for BCBMs including targeting the blood–brain/tumor barrier (BBB/BTB), tumor cells, and tumor-supporting immune cells for concentrated drug release within BCBMs, as well as use of nanoparticles (NPs) for delivering immunomodulatory agents, for inducing immunogenic cell death, or for potentiating anti-tumor T cell responses.

**Graphical abstract:**

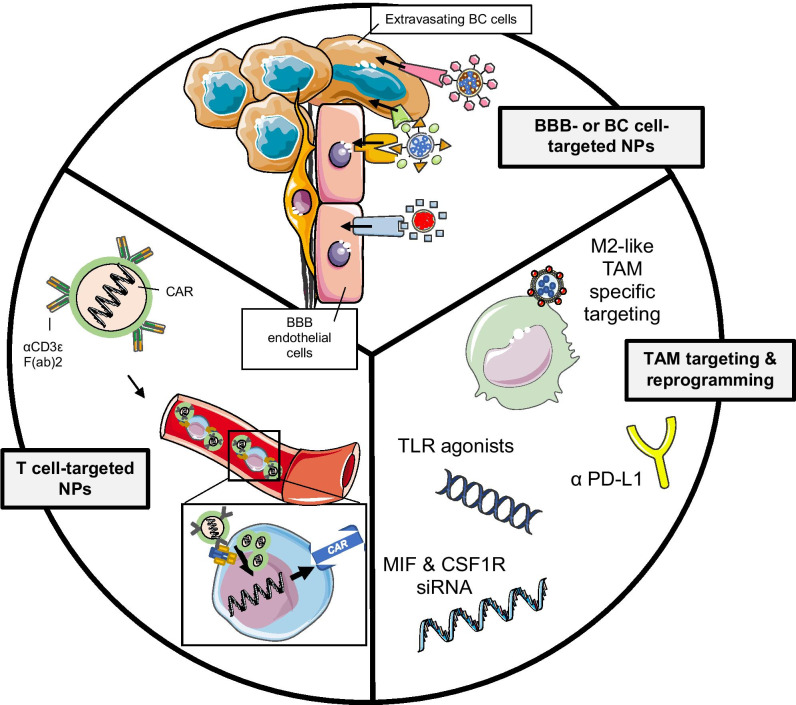

## Introduction

Brain metastases (BMs) are the most common type of intrinsic brain tumor and are associated with an overall poor prognosis, evidenced by median survival < 2 months from diagnosis in untreated patients [1]. Treatment with surgery, chemotherapy, and/or radiotherapy (RT) only extends survival by 4–6 months in many tumor types [2], underscoring the need for new therapeutic strategies. BMs most commonly arise from melanoma, lung, and breast cancers (BCs) [3], the latter of which is the focus of this review. Approximately 30% of all patients with metastatic BC will develop BMs (BCBMs) [4, 5]; however, incidence is more common in patients with triple-negative BC (TNBC) and Her2+ BC subtypes. A recent retrospective study estimated that up to 46% of patients with TNBC and 55% of those with Her2+ BC will develop BCBMs; patients with the latter have better prognosis due to the availability of Her2-targeted therapies [6]. Aside from these targeted therapies, which have restricted delivery across the blood–brain/tumor barrier (BBB/BTB), BCBM treatment is largely palliative and typically involves surgery, whole-brain RT (WBRT), stereotactic radiosurgery (SRS), or combinations thereof [7]. Systemic chemotherapies are rarely used due to poor brain penetration and intolerable side effects from high systemic toxicity [8]. Thus, there is a clear and immediate need to expand and improve treatment modalities for BCBM patients.

Patients typically present with BCBMs 2–3 years after diagnosis of the primary breast tumor, which commonly occurs concurrently or after metastatic spread to the lungs, bones, or liver, which represent other frequent sites of BC metastasis [4]. Metastatic spread generally occurs through hematogenous mechanisms, requiring the BC cells to first traverse the BBB. The BBB, which tightly regulates the transport of molecules from the blood, functions within a larger neuro-vascular unit (NVU) consisting of endothelial cells, pericytes, astrocytes, microglia, and neurons that operate in coupled fashion to monitor and maintain cerebrovascular homeostasis [9]. Upon traversing the NVU, BC cells adopt unique signaling pathways and phenotypes in order to survive in this unique CNS microenvironment [10]. Though initially thought to be minimally infiltrated with immune cells [11], it is now understood that establishment of these brain micrometastases requires reactive, inflammatory components including early infiltration and reprogramming of various immune cells and astrocytes within the brain as a “pre-metastatic niche” (PMN) [12]. The apparent reciprocal communication between metastatic and microenvironmental cells therefore represents an intriguing target for BM treatment. Such approaches, however, have been limited to date by the traditional notion that BCs are immunologically cold or minimally immunogenic [13]. Likewise, the CNS has traditionally been considered an immunologically privileged site, devoid of most peripheral immune cells [11]. Combined with limited penetration of conventional drugs into the brain, patients with BMs are excluded from many clinical trials involving systemic or immunotherapies (ITs), limiting current data related to IT for BCBM treatment. Today, the CNS is regarded as an immunologically *distinct* site under tight regulatory control [14, 15], and there is increasing evidence suggesting that enhancing immune responses against BC cells will greatly improve therapeutic responses and patient survival. Despite the potential of IT, this therapeutic approach faces several specific hurdles for BCBM treatment including significant drug delivery challenges, relatively few targets in BCBM tumors and safety concerns that will continue to hinder translational progress. Nanomedicine, specifically, has the propensity to overcome these barriers and represents a promising strategy for enhancing ITs for BCBMs. Use of nanoparticles (NPs) for intracerebral drug delivery is particularly advantageous due to the ability to engineer and fine-tune NPs for specific biomedical applications. As drug delivery agents, NPs can be decorated with surface ligands to enhance NP targeting to the brain and/or tumor tissues. Further, NPs can encapsulate various therapeutic agents, limiting side effects while enhancing drug stability, and be designed for specific or controlled drug release at the tumor site. Thus, nanotechnology is a potentially valuable tool to improve the delivery, safety, and efficacy of ITs for BCBMs. In this manuscript, we review emerging studies that provide evidence for IT as an effective strategy for BCBM treatment, assess the substantial physiological barriers limiting clinical translation, and highlight the potential of nanomedicine for improving IT effectiveness. We focus on the capability of nanotechnology to improve IT drug delivery and the therapeutic strategies currently under investigation or in clinical development for BCBMs.

## Turning cold tumors hot: emerging immunotherapies for BCBMs

### Mediators of the BCBM immunosuppressive tumor microenvironment

**Fig. 1:**
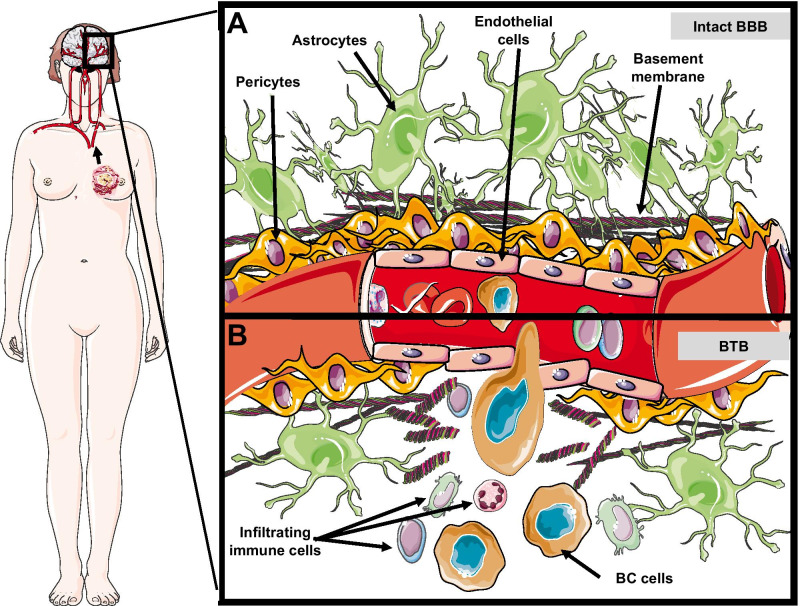
Breast cancer brain metastases (BCBMs) convert the blood–brain barrier (BBB) into a blood-tumor barrier (BTB). **A** breast cancer (BC) cells intravasate at the primary tumor and travel via the circulation to the central nervous system and encounter the intact BBB, consisting of endothelial cells lining the vascular lumen, tightly arranged pericytes, a basement membrane composed of mostly collagen-bundles, and end feet processes projecting from adjacent astrocytes. **B** BC cell extravasation across the BBB induces an acute local inflammatory response involving activation of astrocytes and microglia, promoting BC cells to adopt several signaling pathways for immune evasion and survival. Surviving BC cells progress into BCBMs, resulting in a transformed BTB with disordered endothelial cells, altered pericyte populations, disruption of the basement membrane, reduced astrocytic end feet, and increased recruitment of immature leukocytes. Some graphics in this figure were adapted from Servier Medical Art (CC BY 3.0 Unported)

**Fig. 2:**
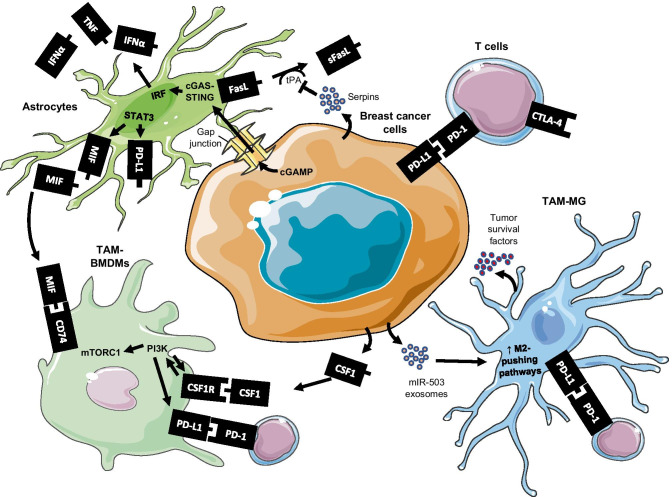
Mechanisms of immunosuppression in the BCBM tumor microenvironment (TME). Breast cancer cells establish reciprocal communication with cells within the premetastatic niche, including resident astrocytes, and establish an immunosuppressed TME conducive for metastatic outgrowth. Tumor-associated macrophages (TAMs) in BCBMs include resident microglia (TAM-MG) and infiltrating bone marrow-derived macrophages (BMDM, TAM-BMDM). Tumor cells secrete factors that promote repolarization of TAMs to M2-like phenotypes that suppress T cell anti-tumor response. *cGAMP* cyclic guanosine monophosphate-adenosine; *cGAS* cGAMP synthase; *CSF1* colony-stimulating factor 1; *CSF1R* colony-stimulating factor 1 receptor; *CTLA-4* cytotoxic T lymphocyte associated protein 4; *FasL* Fas ligand; *IFNα*, interferon-α; *IRF* interferon regulator factors; *MIF* macrophage migration inhibitory factor; *mTORC1* mammalian target of rapamycin complex 1; *PD-1* programmed cell death receptor 1; *PD-L1* programmed cell death ligand 1; *PI3K* phosphoinositide 3-kinase; *sFasL* soluble Fas ligand; *STAT3* signal transducer and activator of transcription 3; *STING* stimulator of interferon genes; *TNF* tumor necrosis factor; *tPA* tissue plasminogen activator. Some graphics in this figure were adapted from Servier Medical Art (CC BY 3.0 Unported)

Cancer cell extravasation and colonization in the brain parenchyma is accompanied by a strong local neuroinflammatory response involving activation of astrocytes and microglia [16]. Metastatic cells—through various factors including cytokine or exosome secretion, and changes in gene expression—recruit and educate immune cells to generate a brain tumor microenvironment (TME) conducive to micrometastasis outgrowth [17]. The BCBM TME consists of several cell types including a variety of immune cells, fibroblasts, endothelial cells, astrocytes, neurons, and tumor cells all physically sequestered by the BBB (Fig. 1A) [17]. As BMs grow, the BBB is remodeled to become a more permeant blood-tumor barrier (BTB) (Fig. 1B), resulting in further infiltration of peripheral immune cells and eventual development of an immunosuppressive TME that shuts off anti-tumor activity [18]. Thus, the key players in establishing and maintaining this TME are largely the targets of current IT strategies. While the growing complexity of the immune system’s role in BCBM is beyond the scope of this review, an overview of the mechanisms by which tumor and immune cells establish an immunosuppressive TME (Fig. 2) is necessary for addressing the therapeutic targeting thereof and is addressed briefly here.

#### Tumor cells

Upon micrometastasis outgrowth within the brain, tumor cells counteract endogenous neuroinflammatory responses by progressively reprograming cells within the TME into immunosuppressive phenotypes, as occurs in chronic inflammatory conditions [19]. Real-time multiphoton laser-scanning microscopy tracking the fate of actively metastasizing cells deep in the live brain has demonstrated that metastatic colonization of the brain happens in perivascular sites along the microvasculature [20]. Known as vascular co-option, this likely occurs due to optimal oxygen and nutrient supply; however, it also enables interaction with infiltrating bone marrow-derived cells from the peripheral circulation [21, 22]. These immature immune cells, together with several tumor-derived factors, play a major role in the formation of a PMN that is conducive to metastatic cell immune evasion, colonization, and outgrowth [19, 23]. The mechanisms governing PMN formation are still poorly understood; however, the ability to reprogram anti-tumor TMEs to a pro-metastatic environment has been proven to be a necessity for micrometastasis progression into macrometastases [24–26]. In one study, it was found that BC cells upregulate genes, including SEMA4D, that protect tumors cells from oxidative stress induced upon crossing the BBB and also disable subsequent immune microenvironment activation [27]. Here, the authors showed that c-Myc, which upregulates free-radical scavenging proteins including GPX1 [28], is overexpressed by metastatic cells to resist oxidative stress caused by activated immune cells. In another study, Wingrove et al. used an optimized RNA-seq pipeline called BM xenograft-RNA sequencing (BMX-seq), an approach that leverages xenograft transcriptomes for distinguishing tumor and stromal cell gene expression, to reveal an upregulation of neuronal differentiation pathways within tumor cells as they adapt to the brain through reciprocal communication with stromal cells [10].

The brain’s reversible upregulation of neuronal differentiation pathways within metastatic cells has also been reported by others. For example, metastasizing BC cells were recently shown to adapt to the unique metabolism of the brain, in part, by mimicking neuronal cells via upregulation of GABAergic genes such as glutamate decarboxylase 1 (GAD1) [29, 30]. This enables metastatic cells to utilize glutamate as an energy source, which is prevalent in the brain. Tumor cells have also been shown to co-opt leukocyte phenotypes to establish BCBMs. For example, tumors upregulate cathepsin S, a protease involved in antigen processing by antigen-presenting cells (APCs), for proteolysis of junctional adhesion molecules and BBB transmigration [31]. Finally, elevated protocadherin7 (PCDH7) expression has recently been detected in patient-derived BCBM samples and in animal models, and has been shown to contribute to BCBM growth by mediating paracrine signaling between tumor cells and astrocytes [32, 33].

#### Neuronal cells

Neurons are the main signaling units of the brain, and though not currently implicated in BCBM progression or associated immune responses, neuronal cell death increases due to reduced vascular perfusion caused by compressive and mechanical stress of growing BCBMs [34]. There has been little exploration into the role of neurons in BM progression; however, protecting neural circuitry will remain an important safety consideration for IT.

#### Astrocytes

Accounting for ~ 50% of healthy human brain cells, astrocytes are glial cells that, similar to macrophages, exist within a spectrum of phenotypic and functional polarization states that aid in diverse homeostatic brain processes including immune responses [35]. Also mirroring macrophages, it appears astrocytes may initially inhibit metastasis but begin to progressively facilitate BCBM growth into macrometastases [36]. Astrocytes can initially prevent metastatic colonization by inducing FasL-mediated tumor cell death [34, 36]. Tumor cells, in turn, escape pro-apoptotic signals through increased serpin expression, which inhibits tissue plasminogen activator (tPA) from astrocytes and prevents active plasmin necessary to convert FasL into sFasL. In contrast to this neuro-protective function, more evidence is mounting that suggests astrocytes promote BCBM colonization and outgrowth [12, 37]. For example, it was shown that gap junctions between tumor cells and astrocytes foster tumor cell proliferation and can protect tumor cells from chemotherapy [38]. It was later shown that tumor cells transfer cGAMP to astrocytes through these gap junctions, resulting in astrocyte cGAS-STING-mediated IRF activation, and subsequent secretion of tumor-supportive IFN-α, TNF, and TGF-α [33] (Fig. 2). These gap junctions are thought to facilitate contact in early metastasis, as only tumor cells at the tumor-stromal border are in direct contact with astrocytes later in metastatic outgrowth [38].

Importantly, astrocytes have also been shown to be integral to the immune response to BCBMs. Recently, it was shown that STAT3+ astrocytes in BCBM clinical samples and mouse models promote BCBM growth via expression of immunosuppressive proteins, including PD-L1, and inhibition of CD8+ T cell infiltration [36]. Further, the authors demonstrated that these STAT3+ astrocytes are the source of macrophage migration inhibitory factor (MIF), which contributes to immunosuppressive TAM phenotypes by binding CD74 receptors expressed by TAMs. The drug silibinin, a STAT3 inhibitor, is being studied for targeting these signaling axes in BMs [39].

#### Macrophages and microglia

Macrophages are a heterogenous group of myeloid-lineage innate immune cells that originate from differentiation of infiltrating bone marrow-derived monocytes (BMDMs) or from proliferation of tissue-resident macrophages that seed distinct organs during embryonic development [40, 41]. Thus, BCBM tumor-associated macrophages (TAMs) include infiltrating BMDMs (TAM-BMDM) and tissue-resident microglia (TAM-MG). The data on these populations is considerably lacking, as most investigations on TAMs in TNBC have focused on primary tumors, or more recently, on BC lung metastases. Sometimes referred to as metastasis-associated macrophages (MAMs), these BC MAMs have been shown to assist in cancer cell extravasation and subsequent growth into lung metastases [42, 43]. A recent RNA-seq study indicated that stromal cells specifically within BCBMs, as opposed to stromal cells in bone or lung BC metastases, exhibit the most pronounced gene expression changes compared to the corresponding normal tissue. Indeed, ~ 54% of the differentially upregulated genes correspond to the gene signature of activated microglia [27], implying that some TAM-targeting strategies may be more suitable for BCBMs than that of other extracranial metastases. While only recently has it been possible to distinguish TAM-BMDM and TAM-MG [40], prior studies reveal that both populations display a range of phenotypic and functional differences based on their context-specific polarization state [44].

Although studies categorizing macrophage populations into M1-like or M2-like phenotypes are overly simplistic, the general consensus is that M1 macrophages are pro-inflammatory and generally inhibit tumor growth while M2 macrophages are tolerogenic and suppress anti-tumor activity [45]. Metastatic cells actively reprogram both TAM-BMDM and TAM-MG to adopt an “M2-like” phenotype, which promotes brain tumor progression through various mechanisms that collectively suppress T cell anti-tumor activity [46, 47]. Indeed, comparison of TAMs in primary tumors versus BCBMs shows significantly higher M2-like gene expression pattern by TAMs in the BCBM TME [44]. A 2010 investigation into the role of microglia in BCBMs confirmed that TAM-MG promote BCBMs by establishing a PMN prior to tumor colonization and act as a guide rail for invasive tumor cells, the latter of which is dependent on Wnt signaling [48]. Importantly, the authors of this study demonstrated that pro-tumorigenic microglia could be repolarized to an anti-tumor phenotype, which underlies therapeutic strategies seeking to re-educate TAMs to a tumoricidal polarization state.

Interestingly, nicotine was recently implicated in TAM-MG skewing to M2-like polarization states in lung BMs [49], necessitating clarification if nicotine has the same effect in BC patients. Nonetheless, these findings challenge a 2006 study reporting that microglia have the capacity to suppress lung BMs [46], which originally posited an anti-tumor role for TAM-MG due to their initial activation and release of pro-apoptotic signals in response to invading tumors cells. Reports from the last decade have clarified these findings and overwhelmingly suggest that both TAM-MG and TAM-BMDM primarily foster BC intracranial metastasis: tumor cells quickly evade microglia-derived apoptotic signals by exploiting tissue damage response pathways to repolarize TAM-MG to tumor-supportive phenotypes [50]. For example, Xing et al. recently demonstrated that metastatic BC cells secrete exosomes containing miR-503 (Fig. 2), which promotes the upregulation of programmed death ligand-1 (PD-L1) and other M2-like markers in TAM-MG, resulting in suppression of T cell proliferation [51]. Collectively, these findings challenge the notion that BCBMs are poorly infiltrated by immune cells—TAMs have since been identified as the most abundant non-tumor cell type in BCBMs [44]—and highlight the potential for therapeutic targeting immunosuppressive TAMs. Several such strategies under clinical and preclinical evaluation are discussed in subsequent sections.

#### T cells

Tumor-infiltrating lymphocytes (TILs), the greatest enforcers of anti-tumor immunity, are present in BCBMs and primarily consist of helper CD4+ and cytotoxic CD8+ T cells [52]. In contrast, bone marrow sequestration often prevents T cell accumulation in primary brain tumors [52]. In fact, in one of the largest retrospective analyses of human BCBMs, CD4+ and CD8+ TIL infiltration was observed in 96% and 98% of cases, respectively, compared to macrophages/microglia observed in 92% of cases [53]. The density of CD8+ TILs was also comparable to that of CD68+ macrophages/microglia; however, there was no correlation between TIL accumulation and overall survival (OS) [53]. This suggests that CD8+ TILs, while capable of trafficking to BCBMs, become exhausted and/or dysfunctional upon encountering the immunosuppressive TME discussed above. In contrast to TIL accumulation, programmed death 1 (PD-1) expression on TILs was independently associated with OS, further suggesting the importance of an activated immune response [53]. PD-1 and cytotoxic T lymphocyte-associated protein 4 (CTLA-4) are both inhibitory receptors expressed by T cells that function as negative feedback loops upon T cell activation in order to maintain immune homeostasis and prevent autoreactivity [54]. While both receptors are essential for maintaining tolerance by counteracting T cell costimulatory signaling under chronic inflammatory conditions, such as in tumors, T cells become exhausted and upregulate these and other inhibitory receptors to inhibit T cell activation, proliferation, survival, and production of IFN-γ, TNF-α, and IL-2 [55]. Consequently, inhibiting these receptors with antibodies has been shown to enhance anti-tumor T cell activity [56] and several clinical trials are underway to evaluate the efficacy of anti-CTLA-4 and anti-PD-1 therapies in BCBMs (Table 1). While there is clear evidence that T cells do infiltrate BCBMs, comparative analyses of intratumoral CD4+ and CD8+ TILs in BC patients show lower accumulation of TILs in BMs compared to extracranial metastases [57] or primary tumors [58, 59], suggesting that ITs that enhance T cell trafficking to BCBMs may also be a valid strategy for enhancing efficacy.

**Table 1:** Immunotherapy (IT) clinical trials underway for BCBMs

Drug/therapy name	IT type	Cancer subtype/inclusion criteria	Phase	# of pts	NCT identifier
Neoadjuvant Ipilimumab and Nivolumab	CTLA-4 and PD-1 inhibitors	Untreated BMs from PDL1+ TNBC, NSCLC, ALK+ lymphoma, EGFR+/ROS− RCC, BRAF− melanoma	II	40	NCT04434560
SRS+Atezolizumab	PD-L1 inhibitor	TNBC BMs	II	45	NCT03483012
Pembrolizumab + SRS	PD-1 inhibitor	> 2 BCBMs	I–II	41	NCT03449238
Nivolumab + SRS	PD-1 inhibitor	BCBMs	Ib	14	NCT03807765
TOPAZ: Tucatinib in combination with Pembrolizumab and Trastuzumab	PD-L1 inhibitor	Her2+ BCBMs	I/II	33	NCT04512261
Tremelimumab ± Durvalumab + SRS or WBRT	CTLA-4 and PD-L1 inhibitor	BCBMs	N/A	28	NCT02563925
Atezolizumab + Pertuzumab + Trastuzumab	PD-L1 inhibitor	Her2+ BCBMs	II	33	NCT03417544
Atezolizumab + SRS	PD-L1 inhibitor	BCBMs	II	45	NCT03483012
GDC-0084 + Trastuzumab	PI3K/Akt/mTOR inhibitor	Her2+ BCBMs	II	47	NCT03765983
Her2/3 DC vaccine, Celecoxib, Pembrolizumab, IFNα-2b, Rintatolimod	DC vaccine, cytokine modulation, PD-1 inhibition	TNBC or Her2+ BCBMs	IIa	23	NCT04348747
Her2/CD3 BATs	ACT	Her2 + leptomeningeal BCBMs	I	16	NCT03661424
Her2-specific CAR T cells	ACT/CAR T cells	Her2 + BMs, any primary	I	28	NCT02442297
Her2-CAR T cells	ACT/CAR T cells	Her2+ BMs, any primary	I	39	NCT03696030
DCVax-Direct	DC vaccine	Lung and breast BMs	I	10 initially, up to 24	NCT03638765
Proteome-based IT	DC vaccine	BCBMs	II/III	60	NCT01782274
Personalized Cellular Vaccine (PerCellVac3)	DC vaccine	Solid tumor BMs	I	10	NCT02808416

https://clinicaltrials.gov/ accessed January 3, 2021

*ACT* adoptive cell therapy; *ALK* anaplastic large cell lymphoma kinase; *BATs* bi-specific armed activated T cells; *BMs* brain metastases; *BCBMs* breast cancer brain metastases; *CAR* chimeric antigen receptor; *CNS* central nervous system; *CSF* cerebrospinal fluid; *CTLA-4* cytotoxic T lymphocyte-associated protein; *DC* dendritic cell; *EGFR* epidermal growth factor receptor; *G-CSF* granulocyte colony-stimulating factor; *Her2/3* human epidermal growth factor receptor 2/3; *HSCs* hematopoietic stem cells; *IFNα*-*2b* interferon alpha 2b; *IT* immunotherapy; *mTOR* mammalian target of rapamycin; *NSCLC* non-small cell lung carcinoma; *ORR* overall response rate; *OS* overall survival; *PB* peripheral blood; *PBMCs* peripheral blood mononuclear cells; *PD-1* programmed cell death protein 1; *PD-L1* PD-1 ligand; *PFS* progression-free survival; *PI3K* phosphoinositide 3-kinase; *pts* patients; *RCC* renal cell carcinoma; *ROS* reactive oxygen species; *SRS* stereotactic radiosurgery; *TNBC* triple negative breast cancer; *WBRT* whole brain radiotherapy

Not all T cells, however, are effectors of anti-tumor immunity. CD4+ regulatory T cells (Tregs) are critical mediators of immune tolerance through suppression of several immune populations, including other T cells and dendritic cells (DCs) [60]. This suppressive function of Tregs is also exploited by tumors as a means to inhibit anti-tumor responses [61]. A 2009 study used ultrasonic tumor aspirates to analyze immune infiltration in brain tumors from 83 patients [62]. The investigators used a suction adaptor to collect aspirated tumor fragments, a technique that is routinely utilized for biopsy of other solid tumors [63] and has since been validated as a sterile biosource for tissue culture studies in gliomas [64], and showed massive Treg infiltration that strongly suppressed TIL function. In fact, they showed that patients with BMs, regardless of the primary tumor, exhibit even higher levels of intratumoral Tregs than that of primary brain tumor patients, signifying Treg recruitment as a general characteristic of BMs. These results suggest that peripheral Tregs in patients with BMs can potentially be targeted to prevent BM accumulation. Further supporting such a strategy, Tregs have also been shown to exhibit increased accumulation as BMs progress [65, 66].

#### Other immune cells

Our understanding of the BCBM TME and its interaction with the immune system is still in its infancy and will require further study to fill the large gaps of data regarding the potential of IT for BCBM treatment. Investigation into other immune cell players in BCBMs has been lacking. To date, there have been no studies investigating the interaction of APCs and TILs that may contribute to a BCBM anti-tumor response, and there is also a scarcity of data concerning the role of neutrophils or natural killer (NK) cells in BCBM pathology [45]. Neutrophils have been shown to accumulate in the BCBM pre-metastatic niche and support tumor cell seeding [67], whereas NK cells were recently implicated in metastatic evasion of immune surveillance, both of which can be targeted to reduce metastatic seeding [68]. Increased investigation into IT for BCBMs, however, will hopefully elucidate the relevance and contribution of these important immune players.

### Current immunotherapy strategies for brain metastasis treatment

Immunotherapeutic approaches can be broadly classified as those that (1) inhibit immunosuppression to release the brakes on anti-tumor immunity and/or (2) enhance immune responses to stimulate anti-tumor activity. Owing to the traditional notion that BCBMs are poorly immunogenic, most clinical trial and preclinical studies to date have assessed IT for melanoma and non-small cell lung cancer (NSCLC) BMs [69]; however, IT studies in the context of BCBMs are beginning to emerge and are reviewed below.

#### Immune checkpoint inhibitors (ICIs)

Checkpoint blockade involves use of ICIs targeting immune checkpoint proteins, including PD-1 and CTLA-4 inhibitory receptors expressed by T cells, as well as PD-1 ligands 1 and 2 (PD-L1, PD-L2) expressed by stromal and tumor cells. Cancer cells co-opt these tolerogenic signaling axes in order to counteract the neuroinflammation induced upon BC micrometastasis [70] and to evade recognition and elimination by T cells. ICIs currently include monoclonal antibodies (mAb's) against PD-1 (nivolumab, pembrolizumab, cemiplimab), PD-L1 (atezolizumab, avelumab, durvalumab), and CTLA-4 (ipilimumab, tremelimumab) (Table 1). Because these BCBM clinical trials have just recently begun, the promise of exploiting these signaling axes for BCBM treatment primarily comes from ICI efficacy reported in BMs originating from lung tumors or melanomas [71]. In a 2019 retrospective study of 271 patients with lung or melanoma BMs receiving SRS, for example, median OS of patients receiving ICIs was 15.9 months compared to just 6.1 months for those who did not receive ICI treatment [72]. Subsequent use of combination therapies increased ICI efficacy, with combination of ipilimumab and nivolumab increasing response rates in melanoma patients to 50–55% [73, 74] compared to that of ipilimumab monotherapy, which had response rates of 16–25% [75]. CTLA-4 and PD-1 are thought to be involved in the early priming and effector phases of T cell activation, respectively, and thus this combined ICI strategy is thought to provide additive efficacy in these patients by modulating temporally distinct components of T cell responses. However, safety remains an important limitation in combined ICI strategies, as 96–97% of patients receiving dual ICI therapy experienced adverse side effects compared to just 68% in nivolumab monotherapy trials [76].

The utility of ICI for BCBMs remains poorly understood due to the exclusion of patients with metastatic BC from most ICI clinical trials if they are also harboring BCBMs. Recently, however, a pilot study of tremelimumab (CTLA-4 mAb) with radiation was completed to determine the abscopal effect and impact on non-CNS disease control in 20 Her2- and 6 Her2+ patients with BCBMs [77]. While the effect of this regimen on BCBM progression was not specifically evaluated, this trial did assess the safety of tremelimumab with trastuzumab (Her2 mAb) and radiation in Her2+ disease, which was well tolerated. Despite the removal of 42% of patients from the study due to rapid non-CNS disease progression or death within 12 weeks, this trial provided important data pertaining to the safety of ICI for patients with BCBMs.

#### TAM and microglia-targeted therapies

In addition to ICI, several promising TAM-targeting strategies are being investigated for BCBMs. The phosphoinositide 3-kinase (PI3K) pathway, which is activated in over 70% of BCBMs [78, 79], plays a central role in the metastasis promoting functions of TAM-BMDM and TAM-MG, in part, by upregulating their expression of immunosuppressive genes including PD-L1 and the colony-stimulating factor (CSF) 1 receptor (CSF1R) [80]. Further, pharmacological inhibition of PI3K in infiltrating TAMs with BKM120 (buparlisib), a class I PI3K inhibitor with good BBB penetration [81], resulted in repolarization of TAMs to a more anti-tumor phenotype and reduced BC infiltration into brain parenchyma [80]. In an earlier study, oral administration of BKM120 in several Her2+ multi-organ metastasis models led to a strong reduction in BCBM tumor burden [82], showing promise for further studies of PI3K inhibition in BCBMs. BKM120 recently completed a phase II clinical trial in combination with the chemotherapy capecitabine in BCBM patients (NCT02000882); however, the results have yet to be published.

Activated PI3K signaling also leads to downstream activation of the protein kinase B (also known as Akt) and mechanistic target of rapamycin (mTOR) pathways, both of which are implicated in the progression of various cancers including BC. There are several drugs targeting crucial components of these pathways; however, only a few are in clinical trials for BCBMs. Everolimus (RAD001) is a mTOR complex 1 (mTORC1) inhibitor with BBB penetrating capability [83] that was approved for advanced HR+ BC patients in combination with aromatase inhibitors in 2012 following the BOLERO-2 trial [84]. Despite the exclusion of patients with BCBMs or CNS-specific responses from the BOLERO-2 trial, everoliumus in combination with trastuzumab and vinorelbine is currently in a phase II clinical trial for Her2+ patients with BCBMs (NCT01305941). Other mTORC1 inhibitors, such as temsirolimus, are also under active investigation in preclinical BCBM models [85]. Dual PI3K/mTOR inhibitors including BEZ235 [86] (NCT014952247, NCT01300962), XL765 [85, 87], and GDC-0084 (NCT01547546) are under preclinical and clinical development in gliomas, and recent data suggests such dual blockade has promise in Her2+ BCBMs [88]. Using orthotopic patient-derived xenografts (PDXs) of Her2+ BCBMs, Ni et al. showed that combined inhibition of PI3K and mTOR with BKM120 and everolimus results in durable tumor regression [88].

There is also an increasing number of TAM-targeting strategies emerging in preclinical studies. The CSF1/CSF1R signaling axis is also a downstream effector of the PI3K pathway that is under active investigation for therapeutic intervention. This pathway is involved in macrophage differentiation and survival [89], and is directly promoted by BC cell secretion of CSF1 [90]. TAM CSF1R signaling, in turn, promotes BC intravasation and invasiveness [31, 91]. Inhibiting this axis with CSFR1 antibodies (RG7155) or receptor tyrosine kinase small molecule inhibitors (PLX5622, PLX3397, BLZ945) inhibits BC tumor growth by reducing and increasing TAM and CD8+ T cell infiltration, respectively, in extracranial BC mouse models [92]. A study evaluating the potential of PLX3397 to prevent melanoma BMs effectively depleted microglia, resulting in reduced BM colonization, holding promise for BCBMs [93]. Interestingly, CSFR1 inhibition was recently postulated to primarily inhibit TAM-BMDMs, as IL-34 serves as an alternate CSFR1 ligand in TAM-MG, likely requiring PLX3397 to be used in a combination therapy. Wnt antagonists represent another TAM-targeting strategy due to observations that TAM-BMDM and TAM-MG help to mediate early BC invasion in the brain in a Wnt-dependent manner [48, 50, 94]. CpG-oligodeoxynucleotides (ODNs) are toll-like receptor (TLR) 9 agonists in clinical trials for various solid tumors [95] and have also been shown to reduce experimental and spontaneous metastases in mouse models of BC [96]. Recently, systemically administered CpG agonists were found to both prevent seeding and reduce growth of BMs by activating microglia [97]. This was performed in metastatic lung and melanoma mouse models but warrants investigation in BC models as well.

#### T cell therapies

**Fig. 3:**
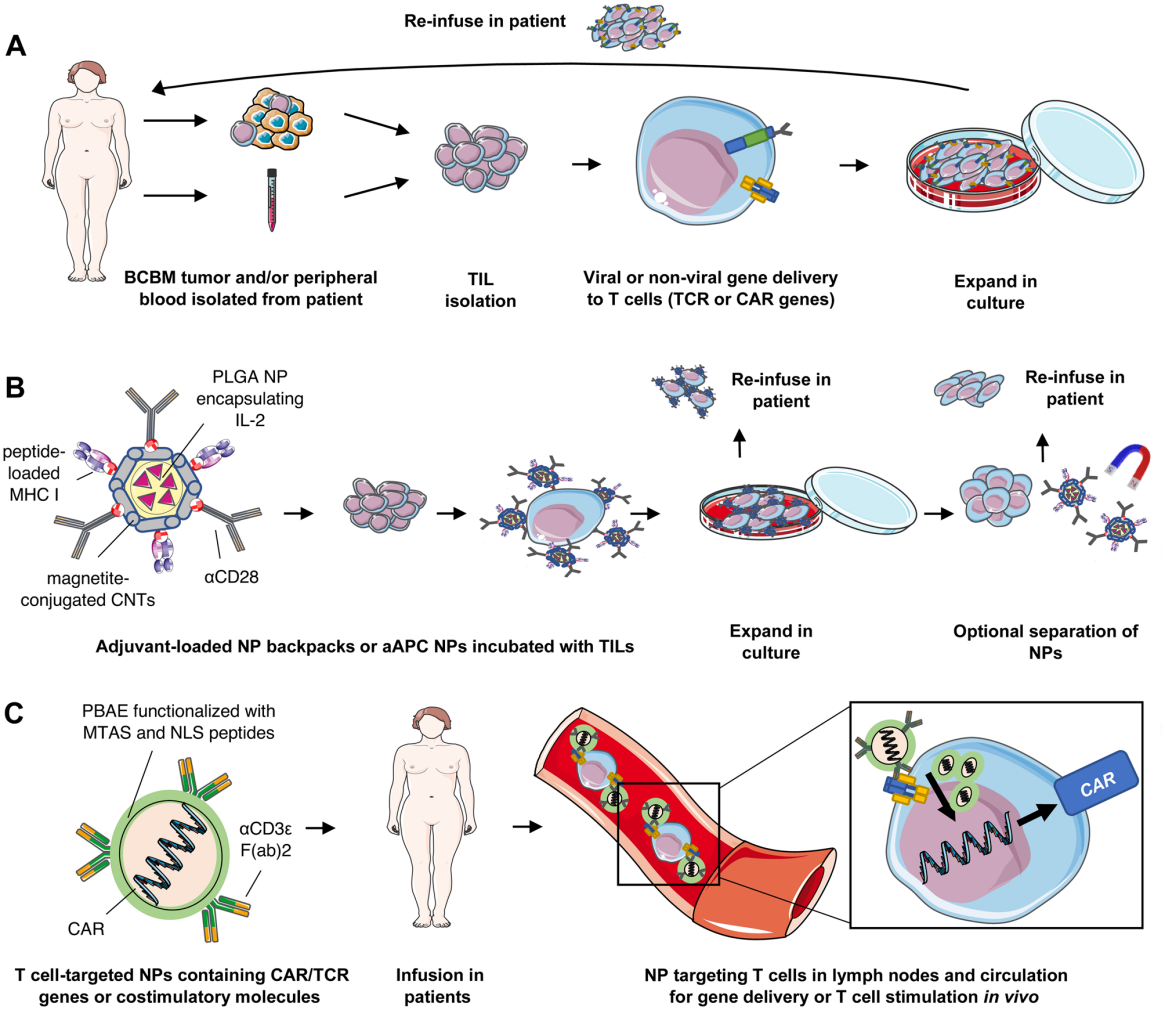
Nanomedicine improves the safety and feasibility of adoptive cell therapy (ACT) regimens. Current regimens for ACT **A** involve (1) isolation of patient T cells from the peripheral blood or the excised tumor, (2) expansion in culture, (3) genetic engineering to induce expression of specific TCRs, (4) selection and expansion of TCR-expressing T cells, and (5) re-infusion into the patient. **B** nanotherapeutics can be used to treat isolated T cells ex vivo, resulting in enhanced anti-tumor activity upon reinfusion into patients. Schematic shows carbon nanotubes (CNTs) functionalized with peptide-loaded MHC I and αCD28 to simultaneously facilitate antigen presentation and co-stimulation to T cells, respectively. Magnetite-mediated conjugation of CNTs to PLGA NPs encapsulating IL-2 further permits paracrine cytokine stimulation while also enabling magnetic separation of NPs from T cells prior to reinfusion in patients. **C** nanotherapeutics can be used to deliver TCR or CAR genes to patient T cells in situ, eliminating the need to culture patient T cells ex vivo. Poly(β-amino ester) (PBAE) NPs are targeted to T cells using a αCD3ε F(ab)2 fragment. Polymers are conjugated to microtubule-associated-nuclear localization (MTAS-NLS) peptides to enhance delivery of cargo to the nucleus. In this example, NPs contain plasmids encoding CAR genes. *aAPCs* artificial antigen-presenting cells; *CAR* chimeric antigen receptor; *MHC I* major histocompatibility complex I; *MTAS* microtubule-associated sequence; *NP* nanoparticle; *NLS* nuclear localization sequence; *TCR* T cell receptor; *TIL* tumor-infiltrating lymphocyte. Some graphics in this figure were adapted from Servier Medical Art (CC BY 3.0 Unported)

IT strategies that do not target immunosuppression involve directly enhancing anti-tumor responses by T cells. Adoptive cell therapy (ACT) involves the ex vivo expansion of autologous TILs or T cell receptor (TCR)-transduced lymphocytes, which are then transferred back into the patient with or without lymphodepletion regimens and/or concurrent interleukin 2 (IL-2) infusion (Fig. 3A). While ACT has been shown to induce durable clinical responses in multiple clinical trials [98], ACT protocols typically require 4–6 weeks of TIL expansion in culture to obtain adequate numbers of reactive T cells for reinfusion [99]. Despite these practical limitations, a retrospective study of 26 patients with melanoma BMs receiving ACT with autologous or TCR-transduced TILs demonstrated that activated lymphocytes could not only traffic to the CNS but can also mediate complete and durable regression of untreated BMs [99]. Based on these and other observations in non-breast BMs, ACT likely holds promise for improving outcomes for BCBM patients. Indeed, in a phase I clinical trial of 23 patients with Her2+ metastatic BC, eight infusions of polyclonal activated T cells transduced with anti-CD3 and anti-Her2 bispecific antibodies (Her2Bi), termed armed ACT (aACT) [100], successfully induced anti-tumor responses and were safe in patients with visceral metastases [101]. This strategy is currently being evaluated using intraventricular administration for patients with Her2+ leptomenigial BCBMs (NCT03661424).

Adoptive transfer of chimeric antigen receptor (CAR)-engineered T cells is another form of ACT that has yielded exciting results for the treatment of solid cancers. Though CAR T cell designs vary, a recent study by Priceman et al. demonstrated Her2-targeted CAR (Her2-CAR) T cells containing 4-1BB intracellular costimulatory domains reduced T cell exhaustion and enhanced proliferative capacity compared to those containing CD28 for costimulation [102]. When evaluating the efficacy of these Her2-CAR T cells in human PDX BCBM models, however, efficacy could only be achieved following local delivery. The authors show that local Her2-targeted CAR T cell therapy results in complete BCBM regression following intracranial delivery, but intravenous (IV) delivery of tenfold greater doses of CAR T cells results in only partial BCBM tumor regression. These results suggest that, despite Her2-targeting, ACT for BCBMs is not quite feasible with systemic drug delivery due to barriers in T cell BCBM trafficking [102]. Regardless, several clinical trials are underway to evaluate the safety, recommended dosage, and efficacy of intraventricularly-administered CAR T cells for BCBMs (NCT03696030, NCT03661424) (Table 1).

#### Vaccines

Cancer vaccination has taken many forms; however, most involve DCs, which are professional APCs that capture and present antigens to T cells for activation. While most studies have focused on the effects of DCs in glioblastoma patients [103], long-term complete remission has been achieved in patients with melanoma BMs following autologous tumor lysate-loaded DC vaccination and RT [104]. With the recognition that BC is indeed an immunogenic disease, multiple BC tumor-associated antigens (TAg’s), including Her2 and mucin 1 (Muc1), are being explored for potential vaccines for patients with extracranial BC tumors [105, 106]. Several clinical trials will help to elucidate the utility of DC vaccines for patients with BCBMs (NCT02808416, NCT01782274), including an ongoing phase I trial investigating the autologous, tumor lysate-pulsed DC vaccine DCVax-Direct (NCT03638765).

#### Radiotherapies to increase immunogenicity

For patients with non-resectable BMs, RT remains the standard of care treatment with WBRT typically indicated for patients with multiple metastases and SRS for those with fewer metastases. RT has long been shown to enhance immunogenicity, and therefore, RT is also being explored as a strategy to sensitize the TME for IT by inducing immunogenic cell death (ICD), which increases tumor mutational burden, major histocompatibility complex I (MHCI) expression, and secretion of inflammatory cytokines [107]. RT ICD is mediated, in part, by a cytosolic DNA pathogen recognition receptor (PRR) capable of initiating anti-tumor responses against tumor-derived DNA. Mechanistically, RT-induced DNA damage activates cGAS, resulting in cyclic GMP-AMP (cGAMP) release and subsequent activation of stimulator of interferon genes (STING) and downstream production of inflammatory cytokines [108]. RT is also particularly of interest in combination with IT due to the abscopal effect, which refers to anti-tumor responses outside of the radiation field. Indeed, the combination of SRS with IT prolongs survival in patients with melanoma BMs [109–111], and several clinical trials, including a pilot studying evaluating CTLA-4 and PD-L1 inhibition with RT (NCT02563925), are ongoing in BCBM patients (Table 1). In a single phase I prospective clinical trial combining RT + ipilimumab in patients with melanoma BMs (NCT01703507), ipilimumab in combination with either SRS or WBRT was safe; however, it failed to demonstrate efficacy of either combinatorial treatment [112]. In that trial, 14 of 16 patients had disease progression and/or had died during the follow-up, demonstrating combined RT + IT is still in an exploratory phase and requires further study to validate the efficacy of this combination strategy.

## Challenges to BCBM immunotherapy

The data summarized above demonstrates the presence of an activated immune response in BMBMs and implies that IT has great potential for treatment of patients with BCBMs. However, despite the approval and use of several IT treatments in various cancer settings, there remain several specific and unique limitations to IT for BCBMs that are summarized below.

### The blood-tumor barrier (BTB) and other challenges to intracerebral IT drug delivery

Drug delivery of most systemic chemotherapeutics to BMs is limited by the BBB, which regulates and severely restricts the movement of molecules from the systemic circulation and into the brain parenchyma. The major cellular components of the BBB include endothelial cells connected via tight junctions, pericytes lining the capillary surface, and astrocytes with their end feet projections (Fig. 1) [9]. The BBB is remodeled to a BTB upon BM outgrowth, consisting of neuroinflammatory endothelial cells and altered pericyte populations, which continues to restrict most cytotoxic agents and drugs from crossing into the brain (Fig. 1B) [113]. Though the BTB is considered to be slightly more permeable than the BBB [114], MRI findings have shown that the BTB does not exhibit elevated permeability in all patients [115, 116], particularly those with Her2+ BMs [117]. The median ratio of trastuzumab levels in the serum and cerebrospinal fluid (CSF) of Her2+ BC patients, for example, was found to be 420:1 [113], which could be improved to 76:1 upon co-treatment with RT. Studies following up on this data reported that trastuzumab-treated Her2+ BC patients have higher incidence of BM development due to a lack of intracranial control of the disease [118]. Endothelial cells comprising the BTB still retain elevated trans-endothelial electrical resistance and large numbers of efflux pumps, which limit paracellular and transcellular drug transport, respectively. As a result, generally only drugs that are lipophilic and have a low molecular weight have efficient transport across the BTB [119], representing a significant barrier to all systemic BCBM therapies, including IT agents.

Even lapatinib, a small molecule inhibitor of Her2 and epidermal growth factor receptor (EGFR) tyrosine kinases that was presumed capable of crossing the BBB, has poor BBB penetration: the average accumulation of lapatinib in BMs was just 10–20% of that of peripheral metastases in mouse models of experimental BMs [120]. Lapatanib has also shown limited activity against human BCBMs [121], underscoring the need for strategies that improve BM penetration. Interestingly, however, combination of capecitabine and lapatinib improved patient response rates [121], suggesting lapatinib acts in a synergistic manner. More recently, evaluation of systemically administration trastuzumab emtanasine (T-DM1) has begun in patients with Her2+ BCs [122–124]; however, the penetration of systemically administered IT remains to studied.

Several other factors in addition to the BTB impede drug delivery to BCBMs. In particular, the brain’s dense, anisotropic, and electrostatically charged extracellular space, combined with elevated interstitial pressure, further limit convective and diffuse drug transport in BMs [125]. Transport is further inhibited by the brain’s glial lymphatic system, which promotes rapid drug clearance from the brain, as well as upregulated expression of multidrug resistance pumps [126]. Such transporters, which include BC resistance protein and P-glycoprotein, contribute to development of resistance, yet another barrier to BCBM drug delivery. Indeed, recent clinical studies have emphasized that even drugs capable of crossing the BTB for Her2+ BCBM treatment still do not provide a significant therapeutic benefit due to resistance mechanisms [127]. Collectively, these limitations to BCBM drug delivery prevent the efficacy of potential ITs and confounds the potential of such strategies. As new strategies to enhance IT and systemic drug delivery emerge, it will also be important to consider how these restrictions to intracranial drug delivery may restrict TIL infiltration into BMs, which has yet to be investigated.

### Limited molecular targets for BCBM IT

The identification of driver mutations and overexpressed proteins in tumors and the subsequent development of drugs targeting these axes have transformed the oncology community over the past decade [128]. While several inhibitors targeting Her2, vascular endothelial growth factor (VEGF), PI3K/mTOR, and EGFR pathways are under investigation for metastatic BC patients, the initial trials investigating their use excluded patients with BCBMs and thus have significantly limited the progress of such strategies for these patients [128]. This has led to a paucity in feasible targets for BCBM therapy, and thus there is a clear demand for identification of more effective targeted therapeutics. Utilization of targeted therapeutics is relevant to BCBM IT strategies for two major reasons. First, targeted delivery of chemotherapeutics or other drugs increases ICD, thereby potentially sensitizing tumors for IT intervention [107, 108]. Indeed, combinatorial approaches utilizing chemotherapies to induce ICD prior to ICI therapy result in superior tumor control than either monotherapy alone [129]. Second, identification of novel targets for BCBMs can be utilized for the development of more efficacious ITs. Tumor cell-targeted NPs, for example, can be utilized as IT drug delivery portals for BCBMs. The utility of such strategies, however, remains limited by the number of actionable targets identified for BCBMs.

### Safety considerations for BCBM IT

The utility and development of IT for many cancers were perhaps most limited initially by substantial safety concerns. High-dose IL-2, an important cytokine for TIL activity, was among the first ITs studied in cancer patients and, aside from its dismal response rates, caused serious adverse effects (AEs), including death [130]. ITs that have followed IL-2 therapy, including ICIs, have only recently begun to incorporate patients with intracranial tumors in safety trials. ICIs generally cause similar immune-related AEs regardless of tumor type, which most commonly affect the endocrine and gastrointestinal systems, skin, and liver [131].

In regard to BMs specifically, investigations into ICI safety have mostly been in patients with melanoma or lung BMs [132, 133]. ICIs as monotherapies have generally been well tolerated in these patients, leading to their approval [69]. Combinatorial ICI treatment, however, has been associated with increased AEs: nivolumab + ipilimumab combination, though more efficacious, was found to cause grade III or IV AEs in 54% of patients, compared to 16% of those receiving nivolumab alone [74]. Despite the discontinuation of ~ 27% of patients, combination ICI was otherwise well tolerated by the majority of patients, suggesting more research is necessary to clarify why some patients experience AEs and others do not. ACT also comes with significant safety concerns, with unexpected organ damage, neurological toxicities and death in early human studies infusing patients with engineered and/or CAR T cells [134]. Toxicities of CAR T cell therapies are numerous and include cross-reactivity against normal cells and immune over-activation, the latter of which can occur through cytokine release syndrome (CRS). The substantial safety considerations of CAR T cells are just beginning to be understood and are reviewed elsewhere [135], but it is also worth mentioning that the complex procedure of engineering human T cells in the lab introduces additional potential safety concerns (Fig. 3A). Several ongoing clinical trials in BCBMs will inform on the safety of these and additional IT strategies for these patients.

## Harnessing nanomedicine for BM immunotherapy

### Nanomedicine for BCBMs

**Fig. 4:**
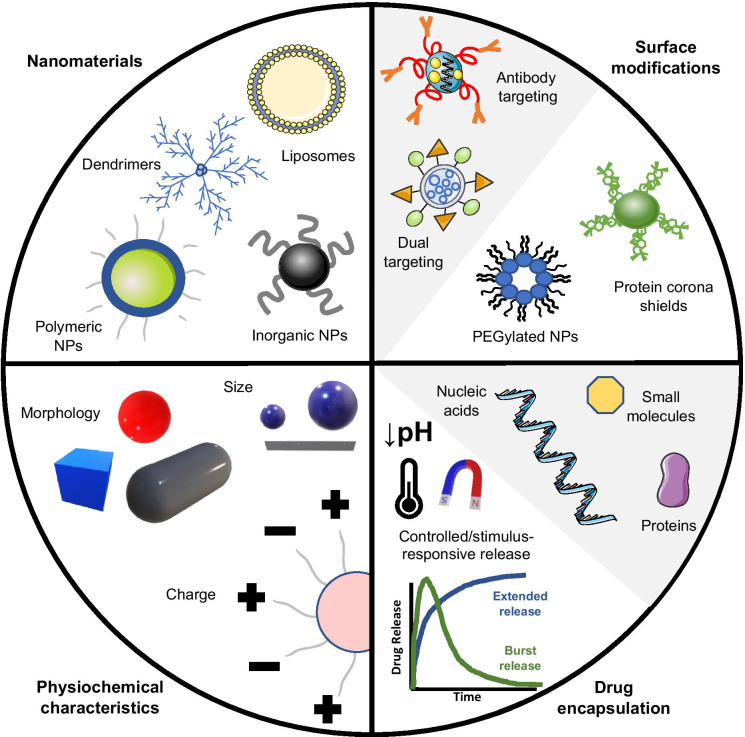
Design and engineering considerations for nanoparticles (NPs). Several types of nanoformulations exist that can be broadly classified as organic or inorganic. Examples of organic NPs include dendrimers, liposomes, micelles and polymeric NPs, while inorganic NPs include gold NPs, iron oxide NPs, and quantum dots. NPs can also be classified based on their physiochemical characteristics, such as NP shape, size, and surface charge. Surface modifications of NPs include use of targeting moieties for specific cellular or tissue localization, as well as modifications that minimize recognition and clearance by the immune system such as PEGylation or use of protein corona shields on NP surfaces. NPs can be designed for delivery of various cargo including small molecules, plasmid DNA, mRNA, and proteins. Encapsulated cargo can be engineered for specific release profiles, and even for controlled release in response to changes in temperature, pH, or application of an external magnetic field

Nanomedicine is a rapidly expanding field and represents a particularly advantageous strategy for enhancing the delivery, safety, and efficacy of ITs for BCBMs. The therapeutic value of nano-oncology resides in the ability to fine-tune physiochemical properties including size, shape, surface charge or targeting of nanotherapeutics (Fig. 4). Further, the characteristically high ratio of NP surface area to volume enables use of multiple surface modifications simultaneously for a given NP, such as conjugation to multiple targeting moieties. In addition, use of NPs can greatly improve drug pharmacokinetics and solubility while simultaneously preventing side effects in patients due to encapsulation of a given drug. The same NP can be modified for encapsulation of different payloads for context-specific use, and furthermore, use of NPs also offers the opportunity for controlled drug release at tumor sites. For example, physiological variables (pH, temperature, redox status) and non-physiological variables (light, ultrasound, electromagnetics) can facilitate the release of concentrated NP payloads within tumors in response to tumor-specific stimuli [136]. Numerous different kinds of NP therapeutics exist, including polymers, micelles, and liposomes, though only two of these are currently in clinical trials for BCBM patients (Table 2).

**Table 2:** Nanotherapeutics currently in clinical trials for BCBMs

Drug/therapy name	Nanotherapeutic type	Cancer subtype/inclusion criteria	Phase	# of pts	NCT identifier
NaI-IRI	Liposome	BCBMs	II	63	NCT03328884
Pyrotinib, Trastuzumab + Abraxane	Albumin NPs	Her2+ BCBMs	II	100	NCT04639271

https://clinicaltrials.gov/. Accessed January 3, 2021

*BCBMs* breast cancer brain metastases; *Her2* human epidermal growth factor receptor 2; *NaI-IRI* nanoliposomal irinotecan; *NPs* nanoparticles; *pts* patients

Generally, NP accumulation in tumors is driven by passive or active targeting. The former generally relies on the “enhanced permeability and retention” (EPR) effect in which leaky blood vessels and dysfunctional lymphatics promote NP delivery to tumors [137], whereas active targeting involves conjugation of tissue- or cell-specific targeting moieties to NP surfaces [138]. EPR-mediated tumor accumulation ultimately led to the development of Doxil and Abraxane, NP formulations of the conventional chemotherapies doxorubicin and paclitaxel, respectively, which are FDA-approved for metastatic BC [139]. It is presently unclear if BMs exhibit an EPR effect for passive nanomedicine accumulation. Recent work by Sinwhwani et al. addressed the mechanisms of NP entry into tumor tissue. Using a combination of transmission electron microscopy (TEM), 3D imaging, computational analysis, and a “Zombie” mouse model that allows for distinction between passive gap and active trans-endothelial transport, this group showed that targeted NPs consistently accumulate in tumors via active trans-endothelial transport mechanisms such as receptor-mediated uptake and vesicular transport or passage through transcellular channels [138]. For BCBMs specifically, NPs are especially poised for improved BTB penetration due to their small size (~ 100 nm) and the potential for reduced neurological and peripheral side effects due to targeting capability and drug encapsulation. Though recently found to be associated with adverse reactions in some patients [140], systemically administered NPs are often engineered with polyethylene glycol (PEG) on their surface to reduce recognition and clearance by the mononuclear phagocyte system (MPS), further enabling intratumoral accumulation [141]. Until recently, NPs were generally designed with such strategies to reduce immune responses. However, NPs are increasingly being studied for use as ITs themselves or in combinatorial treatment strategies. This work is summarized below.

### Nanomedicine-enhanced immunotherapy

While many scientists have published their data and perspectives on either immuno- or nano-therapy for BM treatment individually, there is a considerable lack of original data pertaining to nanomedicine-enhanced immunotherapy strategies in BCBMs specifically. Here, we review current and emerging nanotech strategies for improving IT (nano-IT) for BMs of various primary cancers and their potential for use in BCBM patients specifically. Of these, perhaps the best studied are nanoimmunoconjugates, in which nanostructures are conjugated to antibodies or other targeting moieties to enable more localized IT delivery. Several clinical and preclinical studies are investigating nanoimmunoconjugates targeting (i) the BBB, (ii) tumor cells, (iii) TAMs and myeloid cells, (iv) T cells, and (v) APCs. In addition, use of immune cells as NP carriers and RT to promote ICD are also novel nano-IT strategies in preclinical development.

#### BBB targeting and translocation strategies

Several approaches are under development that harness endogenous transport mechanisms to cross the BBB, including receptor-mediated transcytosis, after systemic administration of NPs [142]. The transferrin receptor (TfR), for example, is of particular interest for intracranial drug delivery due to its high expression on the luminal side of the BBB endothelium [143]. Using three separate tumor inoculation models (intracranial, intracardiac, or intravenous injections), Wyatt et al. demonstrated that systemically administered, pH-dependent mucic acid polymer (MAP) NPs conjugated to camptothecin (CPT, MAP-CPT) (Table 3) can effectively target TfR on the luminal side of the BBB, deliver CPT, and inhibit Her2+ BCBM growth in mice [144]. These NPs also enabled controlled drug release, as pH-responsive portions of the MAP-CPT complex are cleaved due to acidification during transcytosis, releasing CPT into the brain. The same group next assessed the ability of this BBB-targeting MAP delivery system to deliver trastuzumab alone or in combination with CPT [145]. Importantly for IT, which includes ICI therapeutic antibodies, this strategy was feasible for antibody delivery and found that BBB-targeted MAP-CPT-trastuzumab combination NPs result in significantly better tumor control than either therapy alone. Still, NPs with TfR-targeting agents have been shown to remain entrapped in brain endothelial cells or capillaries instead of traveling into the tumor [146, 147], necessitating further investigation into the TfR for BBB translocation.

**Table 3:**
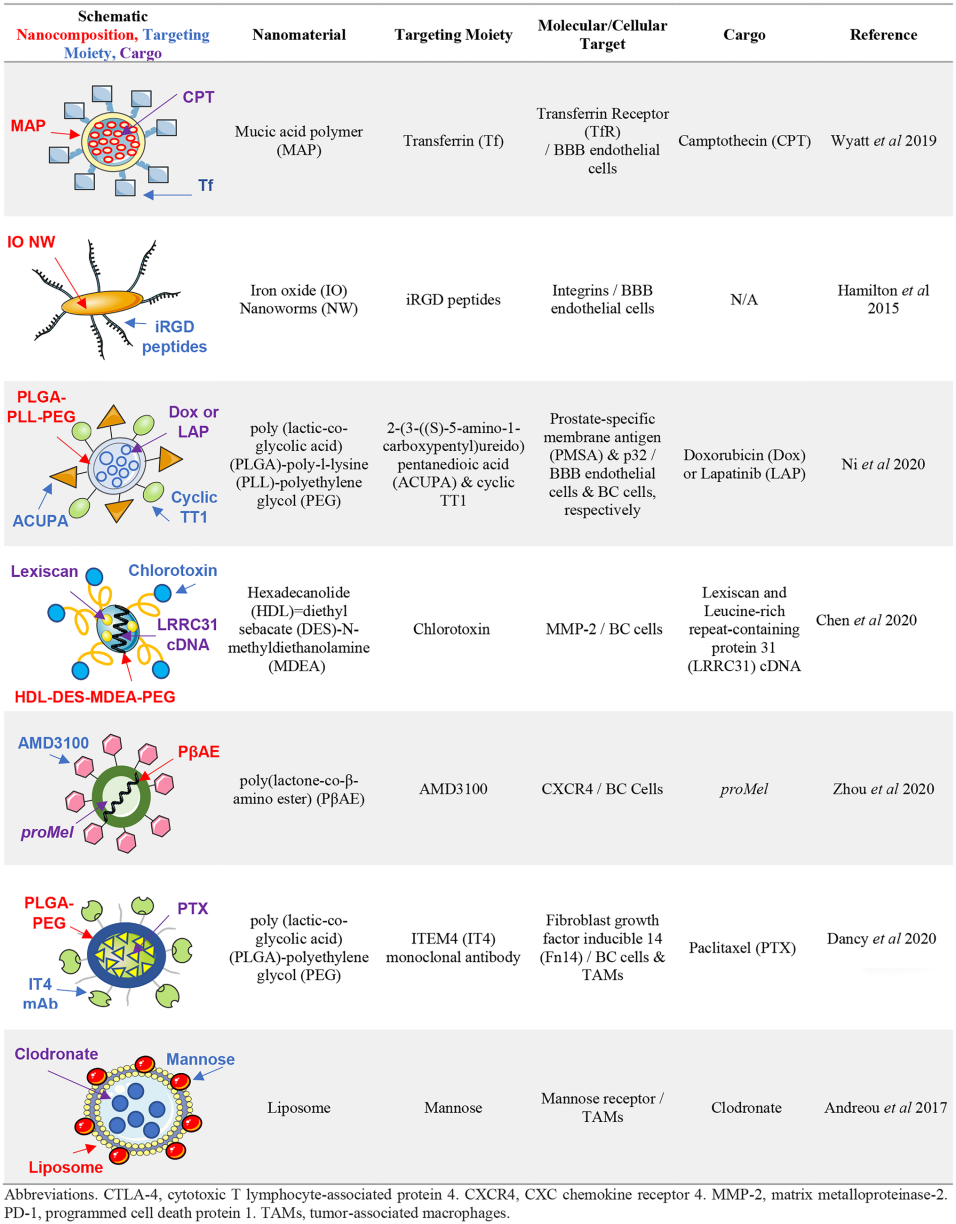
Examples of targeted nanotherapeutics in preclinical investigation for BCBMs

*CTLA-4* cytotoxic T lymphocyte-associated protein 4; *CXCR4* CXC chemokine receptor 4; *MMP-2* matrix metalloproteinase-2; *PD-1* programmed cell death protein 1; *TAMs* tumor-associated macrophages

Another group demonstrated the promise of BBB targeting using iRGD peptides (CRGDK/RGPD/EC), which bind integrins expressed on BBB endothelial cells, and demonstrated that iRGD peptides can be formulated into drug-loaded, tumor-penetrating NPs (Table 3) that are capable of inhibiting BCBM development in two mouse models following a single intravenous injection [148]. More recently, prostate-specific membrane antigen (PMSA) was identified as a potential BBB target, as PMSA is specifically expressed in BCBM-associated endothelial cells, and Ni et al. designed poly (lactic-co-glycolic acid) (PLGA)-poly-l-lysine (PLL)-PEG(PLGA-PLL-PEG) NPs conjugated to ACUPA for PMSA targeting (Table 3) [149]. Furthermore, these NPs were further co-targeted to p32 expressed on BC cells via NP conjugation to cyclic TT1. These dual BBB- and BCBM-targeted NPs not only demonstrated BTB crossing, BCBM inhibition, and prolonged mouse survival, but also the therapeutic benefits of combination therapies. Future BBB-targeting strategies will likely employ further combinatorial approaches. For example, lexiscan-loaded, AMD3100-conjugated, tumor-inducible NPs (LANPs) were recently developed to encapsulate and deliver doxorubicin to BCBM [150]. Lexiscan was used to pharmacologically increase BBB permeability, AMD3100 was utilized to target CXCR4 overexpressed on tumor cells, and a neutrophil elastase (NE)-cleavable peptide was used for controlled drug release at the tumor. The latter is activated by the presence of NE, which is highly enriched in BCBMs. These intricately designed NPs significantly prolonged survival of BCBM-harboring mice, again demonstrating the potential of nanomedicine for fine-tuning drug delivery specifically to BCBMs. To our knowledge, there have been no studies examining the utility of such BBB-targeting for delivering ICIs or other IT agents to BCBMs.

#### Targeting tumor cells

Tumor cell-directed NPs carrying cytotoxic payloads offer the possibility of converting immunologically cold BCBMs into immunoresponsive tumors by debulking the tumor site and concomitantly removing biological barriers to T cell infiltration. Further, direct killing of tumor cells by chemotherapeutics promotes ICD via the release of damage-associated molecular patterns and TAg’s from dying tumor cells, which in turn stimulate innate and adaptive immune responses [151]. In that vein, there has been significant interest in engineering NPs that specifically target proteins overexpressed in BCBMs for preferential drug delivery. Similar to BTB-targeting strategies, exploiting active targeting may provide more opportunity for improving the delivery, safety, and efficacy of IT payloads. Her2-targeted NPs are perhaps the most clinically developed of these drug delivery platforms. For instance, Patil et al. developed a tumor-targeted poly(β-l-malic acid) (PMLA)-based nanotherapeutic platform to deliver trastuzumab to HER2+ BMs in vivo. Here, PMLA was chemically conjugated to (i) trastuzumab to specifically target Her2+ BC cells and (ii) TfR mAb to ensure transcytosis through BBB. The NP-treated mice bearing HER2+ BMs demonstrated significant reduction of Her2 and phosphorylated Akt levels, a downstream indicator of Her2 signaling, thereby increasing the survival time by 57% in comparison to PBS treated mice. Their NP platform was also conjugated to leucine ethylester (LOEt) moieties to promote endosomal escape upon intracellular uptake. MM-302, a Her2-targeted PEGylated antibody–liposomal doxorubicin conjugate, is another NP drug delivery system which has been used to specifically maximize doxorubicin delivery to Her2+ tumor cells. Preclinical studies conducted with MM-302 demonstrated superior efficacy against Her2 overexpressing cancers when administered alone or in combination with trastuzumab or cyclophosphamide, respectively [152–154]. While the clinical trial of MM-302 combined with trastuzumab did not show a significant efficacy difference in comparison to control (NCT02213744) and is terminated [155], tumor deposition data using Cu-MM-302 PET/CT reported the distribution of the nanoconjugates to brain metastasis [156] and demonstrates the ability of these NPs to preferentially and specifically accumulate in BCBMs.

Additional targeting strategies for tumor cells, however, are necessary for BMs without Her2 overexpression, and these targets are beginning to be identified in preclinical investigations. One group recently used CXCR4, which is enriched in BCBMs, to target poly(lactone-co-β-amino ester) NPs to tumor cells (Table 3). These intricately designed NPs were used to deliver an artificial gene, *proMel*, that is activated by MMP-2, which is also enriched in BCBMs [157]. Lapatanib, a dual inhibitor of both Her2 and EGFR, represents another targeting strategy, as EGFR is frequently overexpressed by BCBMs. Wan et al. recently developed lapatinib-loaded human serum albumin NPs (LHNPs) and demonstrated enhanced delivery of the drug to TNBC BMs in animal models, resulting in prevention of metastasis and increased survival time in comparison to clinically approved drug Tykerb [158]. Another group used oleanic acid (OA), which was recently shown to self-assemble into NPs, to deliver paclitaxel to primary breast tumors and BCBMs in mouse models [159]. In this unique approach, the authors previously screened natural compounds capable of self-assembly and selected OA based on its known anti-tumor and anti-viral properties, arguing the anti-tumor activity of the OA carrier itself synergizes with that of the cargo for a combinatorial approach to enhance efficacy. This may open a new direction in BM therapeutics aimed at identifying NP carriers that have endogenous anti-tumor activity. Finally, our group has recently designed paclitaxel-loaded, fibroblast growth factor-inducible 14 (Fn14)-targeted PLGA-PEG NPs (Table 3), which outperformed the clinical paclitaxel-NP formulation, Abraxane, in a mouse model of TNBC growth in the brain [160]. Crucially for BMs, these NPs were specifically designed to exhibit Decreased non-specific Adhesivity to brain extracellular matrix and Receptor Targeting, or DART, characteristics [161, 162]. These NPs target Fn14, which is overexpressed in BCBMs as well as various other solid tumors [163] and thus represents a potential delivery platform for IT agents as well.

#### Targeting and reprogramming immunosuppressive TAMs

Owing to both the prevalence of TAMs and their largely immunosuppressive role in tumors, targeting these key stromal cells in BCBMs by nanomedicine is a highly promising strategy. TAMs are reported to non-specifically take up NPs, and indeed much of the literature involving NP uptake by TAMs involves passive TAM targeting [164]. In studies of extracranial murine tumor models, for example, the majority of Her2-targeted gold NPs were taken up by perivascular TAMs and not by Her2+ cells [165]. TAMs therefore potentially represent a non-selective NP sink, which can be hijacked to deliver agents that promote TAM repolarization to an anti-tumor phenotype. Iron oxide NPs (IONPs), in particular, have been shown to inhibit tumor growth by repolarizing TAMs to pro-inflammatory phenotypes in preclinical BC models [166]. In the case of IONPs, repolarization is based on the NPs themselves as opposed to delivery of an immunomodulatory agent. Recently, tandem peptide nanocomplexes (TPNCs) carrying CpG DNA (TLR9 agonists), which stimulate TAM inflammatory gene expression, were shown to suppress tumor growth and synergize with CTLA-4 inhibition in primary BC models [167], warranting investigation of this strategy for BCBMs.

TAMs have also been studied in the context of active NP targeting for selective cell depletion. Liposomal NPs were recently employed to eliminate TAMs in preclinical BCBM models, wherein intracerebral injections of mannosylated clodronate liposomes resulted in a significant reduction of tumor burden (Table 3) [168]. Another group identified a unique peptide sequence, M2pep, that preferentially binds to M2-polarized TAM populations [169]. This targeting moiety was subsequently used for nanodelivery of CSF-1R siRNA to TAMs in melanoma mouse models and resulted in a significant inhibition of tumor growth [170]. In another study, BG34-10 glucan was identified to mediate specific and active internalization of NPs by primary macrophages [171], and was able to effectively deliver MIF siRNA to TAMs within 4T1 mammary tumors and reduce their MIF expression following systemic administration. While preclinical investigation of M2pep- or BG34-10-glucan-targeted NPs for BCBMs is clearly warranted, these TAM-targeting strategies underscore the increased safety afforded by nanomedicines, as these NPs are internalized by intratumoral TAMs and exhibit minimal uptake by tissue-resident macrophages in the liver, spleen, kidneys, or lungs. NP delivery of ICIs is another potential avenue for TAM targeting; however, this has yet to be inve-stigated in BCBMs.

#### Targeting T cells

Perhaps the greatest limitations to engineered and CAR T cell therapies are the complex in vitro engineering procedures required to produce safe and robust in vivo anti-tumor T cell responses [135]. In these therapies, T cells are (1) isolated from patients, (2) propagated in ex vivo cultures, (3) engineered with vectors to express specific TCRs, (4) selected and expanded in culture, and finally (5) reinfused into the same patient (Fig. 3A). Each of these steps will require further investigation and optimization in order for ACT to be exploited for BCBM intervention. Nanomedicine, however, may allow us to negate some or all of these experimental manipulations (Fig. 3B, C). NPs have been used to manipulate adoptively transferred T cells, with earlier studies demonstrating that adjuvant-loaded NPs conjugated to T cell surface proteins can enhance T cell persistence and function in vivo upon reinfusion into patients [172, 173]. These NPs, termed T cell backpacks (Fig. 3B), can be used to provide exogenous T cell stimulation or to exploit T cell tumor-homing properties as means to deliver therapeutics to tumors [174].

TCRs cluster together in regions referred to as “immunological synapses,” which are required for effective T cell activation upon interaction with APCs [175]. In this vein, several NPs have been proposed for use as artificial APCs (aAPCs) for various purposes. Intricately designed, carbon nanotube-polymer composites (CNPs) have been engineered as aAPCs for optimal expansion of T cells isolated from mice (Fig. 3B) [176]. Carbon nanotubes (CNTs) are first functionalized with multivalent T cell antigens and costimulatory molecules (MHC-I and CD28), followed by CNT magnetite-mediated conjugation to PLGA NPs encapsulating IL-2: the former enables effective T cell stimulation while the latter provides paracrine IL-2 stimulation and the ability to magnetically separate CNPs from T cells following ex vivo expansion and prior to reinfusion. Crucially, adoptive transfer of these T cells back into melanoma-harboring mice resulted in delayed tumor growth, providing proof-of-concept for this approach in other solid tumors. In contrast to this study, NP backpacks need not be removed from T cells prior to reinfusion and can even be used to enable controlled drug delivery in vivo. For example, T cells can be engineered with NP backpacks that, upon reinfusion into patients, release human IL-15 super-agonist (IL-15Sa) in response to TCR stimulation, which allows for higher doses of IL-15Sa to be safely administered and improve efficacy in vivo [177].

Nanomedicine, however, is also making an impact in reducing the need to remove or manipulate patient T cells during ACT regimens in the first place (Fig. 3C). In a groundbreaking study, Smith et al. exploited the intrinsic properties of NPs to deliver leukemia-specific CAR genes to circulating T cells in vivo. Poly(β-amino ester) NPs were targeted to T cells via surface functionalization with anti-CD3e F(ab)2 fragment, the CD19 CAR gene construct was delivered to the nucleus with the aid of nuclear localization and microtubule-associated sequences, and the gene was introduced into DNA via a cut-and-paste mechanism mediated by piggyBac transposase elements flanking the CD19 construct. These NPs were not only capable of reprogramming T cells in situ and inducing efficacy comparable to that of conventional CAR T cell infusions, but they were also able to generate long-lived memory T cells that sustain CAR expression for weeks [178]. Such in vivo engineering and expansion of T cells with NPs reduce safety and efficacy complications associated with ex vivo procedures, and represent a promising new strategy to engineer T cells. Ongoing work has begun to clarify how various NPs can be optimized for gene delivery to human T cells [179].

A final nano-IT strategy targeting T cells involves targeted delivery of immunostimulatory materials. CTLA-4 siRNA has been successfully encapsulated in PEG-block-poly (l-lactide) NPs and able to reduce CTLA-4 expression in T cells in vitro [180]. PD-1 expression by T cells has also been targeted with NPs. In one such study, poly(lactic acid-co-glycolic acid)-block-poly (ethylene glycol) copolymers were designed to selectively target PD-1+ T cells through surface functionalization with CD8 and PD-1 antibodies (Table 3), which effectively targeted CD8+ T cells in the blood, lymphoid tissues, and melanoma tumors [181]. PD-1 antibody fragments serve dual purposes in this system: to target specific T cell subsets and to neutralize PD-1 receptors on T cells. Furthermore, these NPs were co-encapsulated with TLR7/8 agonists, enabling sustained release of these immunostimulants. Both of these studies were performed in mouse models of melanoma; however, NP encapsulation of PD-L1 [182] and PD-1 siRNA [183] has also begun to be studied in primary BC models. For example, Wu et al. investigated use of two inorganic NPs—layered double hydroxide (LDH) and lipid-coated calcium phosphate (LCP) NPs—for PD-1 and PD-L1 siRNA delivery, demonstrating LCPs exhibit better cellular uptake and gene delivery. In contrast to polymeric NPs, lipid NPs typically utilize ionizable or cationic lipids, such as 1,2-dioleoyl-3-trimethylammonium-propane (DOTAP) used in these LCP NPs, which aids in endosomal escape and release of negatively charged material. Such characteristics, combined with use of cholesterol and PEG to improve NP stability, make lipid NPs a preferential delivery platform for nucleic acids [140]. Continued investigation into such strategies will inform on the utility of nano-ICIs in BCBMs, as these were performed in primary BC models.

#### Targeting APCs and nanovaccines

NPs can be engineered to capture released TAg’s following cytotoxic tumor cell death, and subsequently present the TAg’s to local APCs [103, 104]. This augments in situ anti-cancer vaccination and amplifies APC-mediated activation of TAg-reactive T cells. Such NPs, formulated using PLGA-based polymers and surface modifications that enable binding of tumor-derived proteins, have been shown to successfully present TAg’s to APCs and synergize with ICIs in mouse models of melanoma [184]. Such TAg-capturing NPs have been shown to improve IT while also improving the abscopal effect. The utility of such a strategy has yet to be explored for BCBMs; however, combining this strategy with existing BC DC vaccination trials (NCT02808416, NCT01782274, NCT03638765) warrants investigation.

#### Utilizing myeloid cells as NP carriers

Both macrophages and their circulating precursors, monocytes, have an intrinsic ability to take up microparticles and NPs, and similar to that of T cells, intrinsically home to areas of inflammation such as BCBMs. Together with an ability to penetrate the BBB, these properties enable the possibility that such myeloid cells may be utilized for NP delivery to tumors and represent an exciting new direction of the nanotech field. While these strategies first emerged in the context of T cell backpacks, myeloid cell carriers were first reported in Parkinson’s disease models [185] and have since been explored for NP delivery to tumors. Monocyte-derived macrophages were introduced as cellular NP carriers for drug delivery to the CNS due their ability to traffic to sites of tissue damage or inflammation [186]. Ullah et al. recently expanded upon this method and provided proof-of-principle for this targeted drug platform in 3D coculture and spheroid tumor models, exploiting an external alternating magnetic field (AMF) to induce heat locally and trigger temperature-dependent release of drugs from NPs [136]. The authors use macrophages as carriers for their silica-coated superparamagnetic iron oxide NPs (SPIONs), which upon application of the AMF, also simultaneously kills the macrophages. Thus, this drug delivery method induces dual targeting of tumor cells and infiltrating, NP-carrying TAMs. This strategy has also been studied in glioma [187] and other extracranial tumor models [188], as well as using other myeloid cell carriers such as neutrophils [189]. Work in primary tumor models, however, suggests that neutrophils do not take up NPs themselves, but instead transiently disrupt the tumor vasculature to allow for NP accumulation [190]. Cytotoxic CD8+ T cells are also being investigated as potential NP carriers to tumors in preclinical models, becoming backpacks for the NPs themselves [191]. Further studies are necessary to clarify this mechanism and see if this drug delivery method is efficacious in tumors that require BBB penetration.

#### Targeting the immunogenic cell death response via nanotechnology

A rapidly expanding strategy in nano-IT involves use of RT in combination with NP treatment in promotion of ICD. Though studied in immunocompromised mice, iodine NPs (INPs) were recently employed to increase absorption and local deposition of RT energy, which doubled the survival of mice with BCBMs compared to mice treated with RT alone [192]. Follow-up studies in immunocompetent mice will further indicate the translational potential of this strategy in humans. A more advanced example of this strategy is Activation and Guidance of Irradiation by X-ray (AGuIX), a new gadolinium-based NP in clinical trials as a radiosensitizer in BCBM patients in combination with WBRT. AGuIX has contrast-enhancing, cytotoxic, and RT-absorbing properties, the latter of which likely promotes ICD [193]. Additional approaches to combine RT and nanomedicine continue to emerge. Chen et al. recently used a CRISPR screen and identified that leucine-rich repeat-containing protein 31 (LRRC31) sensitizes BCBMs to radiation [194]. After revealing LRRC31’s role as a major DNA repair suppressor, the authors used 60% Hexadecanolide (HDL)-diethyl sebacate (DES)-*N*-methyldiethanolamine (MDEA) PEG polymers (HDL-DES-MDEA-PEG) conjugated to chlorotoxin, a brain tumor-targeting peptide, to encapsulate lexiscan for increased BBB permeability (Table 3). The authors then used these autocatalytic brain tumor-targeted NPs (ABTT NPs) to deliver LRCCC31 cDNA, which increased the survival of BCBM-harboring mice following RT.

#### Hydrogel-based drug delivery systems for IT

A known limitation of systemically administered ITs involves the potential to offset immune homeostasis at off-target sites, thus increasing the risk of fatal side effects [195]. Hydrogel-based biomaterials have been explored in several reports for sustained, localized delivery of various therapeutic agents to tumors [196], providing a foundation to expand such technologies towards delivery of ITs for local immunomodulation. Of notable mention, an intracranially implanted liquid crystal polymer-based microcapsule drug depot has demonstrated the potential of hydrogel-like systems for localized BCBM drug delivery [197]. This non-biofouling implant enabled the codelivery of two clinical chemotherapeutics, temozolomide and doxorubicin, resulting in improved survival of mice with BCBMs [197]. Unlike non-degradable macroscale drug depots, hydrogels are composed of cross-linked polymeric matrices that form hydrated macro- to nano-scale three-dimensional structures [196, 198]. These biomaterials can be engineered for local and controlled release to improve therapeutic efficacy and limit systemic exposure [199]. For cancer IT, hydrogel-based drug delivery systems can also enhance the local efficiency of ICD-inducing therapies by increasing the local concentration and residence time of ICD-inducing drugs and NPs, and can be rendered thermosensitive for rapid in situ sol–gel transitions at physiological temperatures, which makes them useful for local deployment via injections [200].

Not surprisingly, various locally applied hydrogel-based drug delivery systems have shown excellent therapeutic potential for local TME immunomodulation in gliomas [201, 202], holding promise that this strategy may increase the delivery and efficacy of ITs for BCBMs. While investigations into hydrogels for BCBM IT delivery are just beginning to be reported, several groups have begun leveraging hydrogel technology to improve IT drug delivery in extracranial BC models. In primary 4T1 BC tumors, for example, thermal-sensitive Pluronic F-127 polymer-based hydrogels have been used to enhance the delivery and efficacy of liposomes containing Imiquimod, a TLR agonist, in tumor-bearing mice [203]. Similarly, alginate-based hydrogel systems have been used to co-deliver CpG to synergize with systemic ICI treatment, resulting in synergized anti-tumor activity in mice [204]. In this system, hydrogels were also loaded with radio-labeled catalase, enabling highly localized radiation, alleviation of tumoral hypoxia, and complete tumor regression in mice. In addition to TLR agonists, ICIs have also been incorporated into hydrogel delivery systems. Alginate polymer-based hydrogels have been used for dual delivery of two FDA-approved drugs—PD-1 mAb and the COX2 inhibitor celecoxib—in mice harboring 4T1 BC lung metastases [205]. This hydrogel-based system enabled local sustained release of the two drugs, resulting in high drug concentrations in the TME and peripheral circulation, a reduction of tumor burden, and significantly improved local and systemic anti-tumor immunity by mobilizing effector T cells and reducing Tregs and MDSCs in the TME.

Employment of hydrogels for IT delivery also has the potential to increase the immunogenicity of tumors. For example, hydrogel-based systems can be used to locally deliver encapsulated TAg’s, which can increase the efficacy of locally applied ITs. Tumor-derived TAg’s have been encapsulated with indocyanine green (ICG) and JQ1, a drug known to suppress PD-L1 expression, in injectable hydrogels containing the tumor penetrable peptide sequence Fmoc-KCRGDK. The cellular release of this molecular cargo upon near-infrared (NIR) illumination serves both as a vaccine node and as a reservoir for local ICI [206].

Hydrogel-based systems can also be used to improve T cell responses and therapies. For example, a nanocomposite system composed of macroporous alginate hydrogels modified with the collagen mimicking GFOGER peptide and loaded with silica microparticles that release IL-15–IL-15Rα complexes has been described. The GFOGER peptide aids in binding TILs to the scaffold, and the microparticles also had anti-CD3, -CD28, and -CD137 surface modifications to enhance TIL co-stimulation. Altogether, this hydrogel nanocomposite system enabled the expansion of transplanted CAR T cells in mouse models of BC resection and disseminated ovarian metastases, leading to a reduction in tumor relapse compared with that observed following systemic or local T cell infusions alone [207].

Taken together, these preclinical studies indicate that hydrogel-based local delivery of ITs, particularly in combination with other therapeutic strategies, may improve local IT efficacy in solid tumors and thus may be of benefit for treating BCBMs.

### Limitations of nanotech for immunotherapy

Despite the promise of nano-IT, there are several limitations that need to be addressed. For example, NPs are still subject to clearance mechanisms from the circulation. Phagocytes comprising the MPS take up NPs and promote clearance by the liver and spleen [140]. NPs can be designed for decreased interaction with the MPS, such as through PEGylation, but the MPS also reveals clearance as a major flaw to strategies seeking to use myeloid cells as NP carriers, as these phagocytes mediate clearance through these tissues. NP interactions with the MPS are also the source of another potential limitation of nanotherapeutics, as these phagocytes can trigger adverse immune responses. PEGylation has recently been shown to cause anaphylaxis in small numbers of patients, which may be mediated in part by anti-PEG antibodies [140]. Investigation into the nuances of the interaction between the MPS and any given NP formulation will be important for its translational potential and negating the risk of adverse allergic reactions in clinical trials. Another confounding issue for targeted nanotherapeutics is the formation of protein coronas upon exposure to serum proteins in the systemic circulation, which can interfere with NP targeting. Though the mechanisms regulating NP interactions with serum proteins are just beginning to be understood, strategies such as cloaking NPs with protein corona shields are under investigation [208]. A final lingering barrier to the translational potential of nano-IT involves scale-up difficulties. NP production strategies utilizing bottom-up processes will need to be scaled up by pharmaceutical companies that predominantly rely on top-down methods [209]. Therefore, maintaining the desired NP formulation characteristics will be especially important for translating nano-IT.

## Conclusions and future directions

The introduction of IT has revolutionized cancer treatments, and despite the exclusion of most BM patients from many early clinical trials, there is expanding and compelling evidence that ITs provide a therapeutic benefit to BCBM patients. As discussed here, these IT strategies remain subject to several crucial barriers for BCBM efficacy including poor BBB permeability, significant safety uncertainties, drug resistance, and an inability to overcome immunosuppressive thresholds to mount an anti-tumor response. Nanomedicine, which has greatly improved intracranial drug delivery and tumor targeting of BCBMs, is particularly poised to overcome these barriers to IT utility. NPs afford the ability to control drug release, which can substantially reduce side effects of a given drug while simultaneously increasing its safety profile. Additionally, fine-tuning of NP physiochemical properties enables targeting of NPs to specific tissues or cell types, further reducing risk of non-specific toxicities. Nanomedicine may therefore have the capability to release the full potential of ITs for BCs in general, but particularly once it has spread to the brain.

As more progress is made in understanding the BCBM tumor-immune microenvironment, new opportunities for IT and nanomedicine are likely to emerge. NPs targeting other immune cell types will likely appear in future BCBM studies. For example, MDSC depletion via polymer NPs loaded with 6-thioguanine has been studied in the context of melanoma [210]. The communication between immune populations and metastatic BC cells is just beginning to be explored; likewise, the interaction of NPs with immune populations is just beginning to be understood. Filling these gaps will greatly improve ITs, nanotherapies, and nano-IT. Still, progress in the field will depend on inclusion of BCBM patients in future clinical trials, as several previously ongoing IT clinical trials have been terminated as a result of low accrual (NCT02669914, NCT00227656, NCT01132664). As the field of nanotechnology expands, potentially leading to the identification of new classes of NP carriers harboring endogenous anti-tumor activity, new directions for BCBM IT will emerge.
